# Case Report: Neurological examination, computed tomography, and magnetic resonance imaging in South American camelids and a dromedary

**DOI:** 10.3389/fvets.2026.1798164

**Published:** 2026-04-30

**Authors:** Marlene Sickinger, Daniela Farke, Meike L. Schmidt, Martin J. Schmidt

**Affiliations:** 1 Clinic for Ruminants and Herd Health Management, Justus-Liebig-University, Giessen, Germany; 2 Department of Veterinary Clinical Sciences, Neurosurgery, Neuroradiology and Clinical Neurology, Small Animal Clinic, Justus-Liebig-University, Giessen, Germany; 3 Institute for Veterinary Pathology, Justus-Liebig-University, Giessen, Germany

**Keywords:** alpaca, central nervous system, diagnostic imaging, llama, tylopods, veterinary

## Abstract

**Introduction:**

South American camelids (SACs) and dromedaries presenting with neurological abnormalities are increasingly common in veterinary practice. Advanced diagnostic imaging techniques, such as computed tomography (CT) and magnetic resonance imaging (MRI), are typically available only in specialized veterinary clinics, and their feasibility is often limited by the animal’s size. We aim to describe CT and MRI findings in a cohort of SACs and dromedaries diagnosed with neurological diseases.

**Methods:**

Medical records were retrospectively reviewed for SACs and dromedaries that underwent CT and MRI of the brain or spinal cord between 2010 and 2025. Fourteen alpacas, two llamas, and one dromedary met the inclusion criteria.

**Results:**

Intracranial diseases included trauma (*n* = 1) and inflammatory conditions, such as abscess formation (*n* = 1), and intracranial mycotic granuloma (*n* = 1). Otitis externa and interna with intracranial extension were observed in five cases. Spinal cord disorders included discospondylitis (*n* = 5), vertebral subluxation (*n* = 1), and trauma-associated intramedullary lesions (*n* = 2).

**Conclusion:**

CT and MRI are valuable tools for diagnosing and characterizing neurologic diseases of the central nervous system in SACs and dromedaries and should be part of the diagnostic work-up when feasible.

## 1 Introduction

Camelids have gained popularity in private ownership worldwide, particularly South American camelids (SACs) (1). Therefore, research addressing their medical challenges has increased. A wide range of neurological diseases has been reported in SACs and other domestic tylopods (2–6). Unfortunately, these diseases are frequently fatal, with definitive diagnoses often requiring post-mortem examination. Computed tomography (CT) and magnetic resonance imaging (MRI) provide detailed visualization of central nervous system (CNS) structures, and their use has significantly advanced the *in vivo* diagnostic capabilities in veterinary neurology.

While CT and MRI are well established in the diagnosis of neurologic diseases in large animals, including horses (7) and ruminants (8, 9), their application in camelids remains limited. Published reports of CNS imaging in SACs are confined to isolated case studies (6, 10, 11), and no systematic imaging-based characterization of neurologic disease in these species exists. To address this gap, in this retrospective study, we aimed to describe CT and MRI findings in camelids presenting with neurologic diseases across a 15-year period at a single referral center, and where available, match imaging findings with post-mortem results.

## 2 Materials and methods

The medical record databases of the Justus-Liebig-University Giessen were searched for camelid patients with suspected neurological diseases who underwent CT and/or MRI diagnostics between 2010 and 2025. The search included records from the Small Animal Clinic Department of Veterinary Clinical Sciences (Neurosurgery, Neuroradiology, and Clinical Neurology) and Clinic for Ruminants with Herd Health Management. Inclusion criteria encompassed all SACs and dromedaries (i.e., tylopods) that presented with neurological signs and had undergone CT and/or MRI of the brain or spinal cord.

Magnetic resonance imaging examinations were performed using a 1.5 (Phillips Intera Gyroscan, Philips Healthcare, Hamburg Germany) or 3.0 (Siemens Magnetom Verio, Erlangen Germany) Tesla high-field scanner. The MRI regions scanned in live animals included the brain (*n* = 6), cervical spine (*n* = 4), thoracic spine (*n* = 4), and lumbar spine (*n* = 6).

Coil selection and imaging protocols differed depending on the anatomical region. For brain studies, dedicated head or neck coils were used. Cervical spine imaging was conducted with a neck coil, while thoracolumbar and lumbosacral spine imaging used spine coils. Brain MRI protocols included sagittal and transverse T2-weighted (T2-W) images (Turbo Spin Echo, TR 2,900 ms, TE 120 ms, slice thickness 3 mm), as well as transverse fluid attenuated inversion recovery (FLAIR) and T1-weighted (T1-W) pre- and post-contrast sequences.

Computed tomography was performed using a 16-slice helical scanner (Brilliance Philips, 120 kV, 200 mAs, slice thickness 1 mm), with image reconstruction in bone and soft tissue windows.

For each case, clinical presentation, results from blood work or additional diagnostic testing (e.g., cerebrospinal fluid [CSF] analysis), diagnostic imaging findings, and necropsy results were reviewed. Computed tomography and MRI interpretations were performed by a European College of Veterinary Neurology board-certified veterinary neurologist (DF). Clinical data and additional diagnostics were reviewed by a nationally certified specialist in bovine and small ruminant medicine (MS). Necropsy findings were analyzed by a resident of the European College of Veterinary Pathologists (MLS) under direct supervision of a diplomate. For the retrospective clinical data analysis, no Institutional Animal Care and Use Committee approval or other approval was needed. Owner consent for diagnostic procedures and use of anonymized data was obtained. Descriptive statistical analyses were conducted by the corresponding author.

## 3 Results

Seventeen animals met the inclusion criteria, comprising 14 alpacas, two llamas, and one dromedary. Male and female animals were almost equally represented, with nine males and eight females. The median age of the examined alpacas was 2.58 years (range, 0.13–19.25 years). The two llamas and dromedary were 2.8, 1.25, and 11.7 years old, respectively. All animals underwent imaging under general anesthesia. Due to fatal prognoses, 10 animals were euthanized, three died during therapeutic attempts, and four were discharged from the clinic. Necropsies were performed on five alpacas, two llamas, and one dromedary.

### 3.1 CT/MR-imaging and techniques

All examinations were conducted on live animals under general isoflurane anesthesia. Food and water were withheld for 12 h prior to anesthesia. An inflatable tube was placed in the esophagus and compartment C1 to minimize the risk of gas accumulation, bloating, regurgitation, and aspiration of compartmental contents. Animal positioning was determined by balancing optimal imaging conditions and anesthesia safety.

### 3.2 CT/MRI findings

Based on their final diagnoses, all included camelids were assigned to one of four pathomorphological groups. A summary of findings is presented for each group, followed by detailed case descriptions, highlighting the characteristic disease processes observed. An overview of case presentations is provided in Table 1.

**Table 1:** Overview of case presentations (1—9) with case number (Nr.), history, clinical signs, and neuroanatomical localization (NL).

Nr.	Signalement	Clinical signs & neuroanatomical localization	Advanced diagnostic imaging	Other	Diagnosis	Outcome
1	2-month-old female Huacaya-alpaca cria	History: 5 weeks progressive ataxiaGeneral examination: UnremarkableNeurological examination: Progressive proprioceptive ataxia and ambulatory tetraparesisNL: C6-Th2	MRI: C5 and C6 endplates with irregular shape and mild disc protrusion. Surrounding tissue heterogeneous T2- and STIR-hyperintense signals. Mild contrast enhancement in T1-weighted images in the cranial aspect of C6 and the subvertebral musculature.	Hematology: WNL Biochemistry: WNL, Serum amyloid A at 5.7 µg/mL (reference <2.7 µg/mL)	Discospondylitis with associated paraspinal myositis was suspected.	Euthanasia
2	A 2.5-year-old female alpaca	History: 3 weeks of reduced general condition, diminished appetite, intermittent recumbencyGeneral examination: Cachexia with BCS 1/9Neurological examination: Paraplegia with hyperreflexia of the spinal reflexes in both hind limbsNL: Th3-L3	CT: Irregular osteolytic endplates of the Th10/Th11 intervertebral disc space and a paravertebral mass lesion with contrast enhancement at the same level.MRI: Endplates Th9 up to Th11, as well as the surrounding tissue were hyperintense in T2 and STIR with irregular shape of the endplates. Mass lesion within the paravertebral muscles. Spinal cord compression at Th10/11 by T2-hyperintense material. Moderate contrast uptake in T1-weighted images within the endplates, vertebral canal and epaxial muscles.	Hematology: Leukocytosis at 26 × 10^9^/L (reference 8–21.4 × 10^9^/L) and PCV of 0.2 l/L (reference 0.27–0.45 l/L). Biochemistry: WNL. Bacterial culture: NegativeNecropsy: Severe chronic purulent discospondylitis and osteomyelitis. Osteolysis and reactive bone proliferation observed at thoracic vertebrae T10 and T11.	Discospondylitis with secondary spinal cord compression and the presence of a paravertebral abscess with secondary myositis of the epaxial musculature in Th9–Th11	Euthanasia
3	1-year-old female llama	History: Reduced general condition, disorientation, ataxia and oral bleeding General examination: Heart rate of 88/min, respiratory rate of 36/min, body temperature of 37.6 °C.Neurological examination: Obtundation, generalized vestibular ataxia to the left. Reduced proprioception in all limbs. Left-sided head tilt, horizontal nystagmus with fast-phase saccades to the left, right-sided facial paralysis. Pupillary reflexes and nasal sensation decreased NL: Multifocal intracranial lesions involving brainstem and forebrain.	MRI: A focal, well demarcated, extra-axial mass lesion at the level of the left temporal and parietal region causing a severe mass effect and midline shift to the right and caudal cerebellar herniation.CT: Similar findings	Hematology: Leukocytosis 27.4 G/L (reference 8–21.4 × 10^9^/L), PCV of 0.24 l/L (reference 0.27–0.45 l/L). Biochemistry: (7.4 mmol/L), hyperglycemia (12.5 mmol/L). Parasitological examination: 200 nematode eggs/g feces 200 coccidian oocysts/g feces Microbiological cultures (CSF and abscess material): Negative	Abscess in the left cerebral hemisphere resulting in a profound mass effect on the surrounding brain parenchyma	SurgeryEuthanasia
4	14-month-old female alpaca	History: Diagnosis of chronic external otitis rightGeneral examination: Chronic purulent discharge from the right external acoustic meatus.BCS 4/9Neurological examination: Unremarkable	CT: Right external acoustic meatus, tympanic bulla, and inner acoustic meatus filled with heterogeneous soft tissue-attenuating material. Thickened medial bulla wall with multiple osteolytic lesions. Post-contrast imaging with marked enhancement of the bulla lumen and osseous remnants. Enlargement of right medial retropharyngeal lymph node	Microbiological culture (material): *Bacillus* spp., *Enterococcus gallinarum, Rhodococcus hoagie, Trueperella pyogenes*Necropsy: Chronic suppurative otitis media with dorsal bulla empyema	Right-sided chronic otitis externa and media, with secondary hyperostosis of the *os tympanohyoideum*	Lateral bulla osteotomy Euthanasia
5	3-year-old Huacaya alpaca gelding	History: Decreased activity, self-imposed isolation, frequent recumbency, and reduced appetite General examination: Respiratory rate of 60/min.Neurological examination: Non-ambulatory tetraparesis, reduced proprioception and right-sided pleurothotonus. Palpebral reflex and menace response bilaterally absent. Bilateral jerk nystagmus with fast phase to the left and changing to positional vertical nystagmus. Horner syndrome, on the right side. NL: Brainstem with right-sided lesion of the central vestibular system	MRI: Right-sided T2- and FLAIR hyperintense heterogeneous material within the external ear canal, middle ear cavity and inner ear with intracranial extension and formation of an extra-axial mass lesion at the level of the right *os petrosum*. Secondary ventriculomegaly with signs of increased intracranial pressure. Focal contrast enhancement in T1-weighted images within the meninges		Chronic bacterial otitis externa, media, and interna with otogenic abscess formation extending into the neurocranium	Euthanasia
6	11.75-year-old female dromedary	History: 3-week progressive gait and behavioral abnormalities General examination: Mucous membranes mildly cyanotic.BCS 2/9Neurological examination: Altered mentation and head pressing, circling to the right, right-sided pleurothotonus. NL: Right forebrain lesion	MRI: A focal, well demarcated, space-occupying, rounded, heterogeneous, extra-axial mass at the level ventral and caudal to the frontal sinus with extension into the cranial cavity, resulting in a severe mass effect on the forebrain. The occipital lobes were displaced into the caudal cranial fossa, compressing the midbrain. The mass had T2- and T1-hyper- and hypointense regions of varying dimensions.	CSF (lumbar): WNLNecropsy: Well-circumscribed, partially mineralized mass (7 cm in diameter) causing marked compressive atrophy of both frontal lobes. Histopathological analysis: granulomatous inflammatory process with extensive necrosis and dense aggregates of fungal hyphae, consistent with a mycotic granuloma. Microbiological analysis: *Aspergillus fumigatus, Streptococci, Serratia marcescens, and Rothia nasimurium*	Intracranial extra-axial fungal granuloma with secondary bacterial infection	Euthanasia
7	3-year-old male llama	History: ataxia, with transient improvement on steroidal anti-inflammatories and attempted CSF puncture L5/6.General examination: UnremarkableNeurological examination: Proprioceptive hindlimb ataxia, reduced proprioception in both hindlimbs, and mildly decreased patellar reflexes. NL: L4-6	MRI: At the level of L5/L6, a small, ill-defined, straight intramedullary T2-hyperintense lesion was visible	Necropsy: Wedge-shaped lesion extending across nearly the entire diameter of the caudal lumbar spinal cord.Histopathological analysis: Focal myelomalacia with associated edema, hemorrhage, and phagocytic removal of necrotic debris.	Iatrogenic spinal cord injury following attempted CSF collection with secondary myelomalacia.	Progression and euthanasia
8	6-month-old male Huacaya alpaca	History: Acute cervical injury after being trapped in a pasture fence General examination: Respiratory rate of 48 breaths/minNeurological examination: Non- ambulatory tetraparesis with increased muscle tone and mildly exaggerated reflexes in all four limbs. NL: C1–C5	CT: Fracture through the cranial endplate of the second vertebra with a dorsal displacement of the caudal fragment and a secondary step formation within the spinal canal, resulting in severe spinal cord compression		C2 fracture with severe spinal cord compression	Euthanasia
9	4-month-old female alpaca	History: Slowly progressive tetra paresisGeneral examination: UnremarkableNeurological examination: Ambulatory tetraparesis with proprioceptive deficits in all limbs, normal spinal reflexes. Kyphosis of the lower cervical spine. NL: C6-Th2.	CT: Severe kyphosis of the lower cervical vertebral column. Vertebral bodies C7 and Th1 shortened in length, and the rostral extremity was wedge-shaped, causing malarticulation with the cranial vertebral segment. Malarticulation with secondary subluxation and narrowing of the vertebral canal. Absent vertebral arches of C7 and TH 1. CT-myelography: Contrast passed readily from the cisterna magna until C5. Over C5, severely diminished dorsal contrast column with reappearance for a short distance over C7. At the caudal aspect of C6, the ventral contrast column was no longer visible and the dorsal column reappeared.	Hematology: WNLbiochemistry: WNL	Congenital spinal malformation with compression of the lower cervical spinal cord	Euthanasia

Findings from computer tomography (CT), magnetic resonance imaging (MRI), and additional diagnostics are listed (WNL = within normal limits). Diagnosis and outcome are presented for each case.

#### 3.2.1 Group a: inflammatory diseases of the brain and spine

Inflammatory conditions affecting intracranial structures, the spinal column, and adjacent soft tissues were diagnosed or suspected in 11 camelids. Computed tomography/ Magnetic resonance imaging findings included discospondylitis with or without perivertebral involvement (*n* = 4), physitis (*n* = 1), inflammation and fusion of the dorsal spinal processes (*n* = 1), otitis and bulla empyema (*n* = 4), intracranial abscess formation (*n* = 1), and intracranial mycotic granuloma (*n* = 1).

In three of the four discospondylitis cases, biopsy samples were collected postmortem from the infected sites for microbiological evaluation. Microbiological culture was positive in one previously published case (10).

##### 3.2.1.1 Case 1

A 2-month-old female Huacaya-alpaca cria was referred for progressive ataxia that had developed across approximately 5 weeks prior to presentation. General clinical examination was unremarkable. Neurological examination revealed a mild proprioceptive ataxia of all limbs but normal proprioception, as well as intact spinal reflexes in all limbs and normal cranial nerve testing. However, the cria exhibited reluctance to move, staggered during palpation, and showed marked cervical pain during passive manipulation. Cervical muscle tone was generally increased. Neuroanatomical localization included the cervical spine with the segments C1-C5 and C6-Th2.

**Figure 1:**
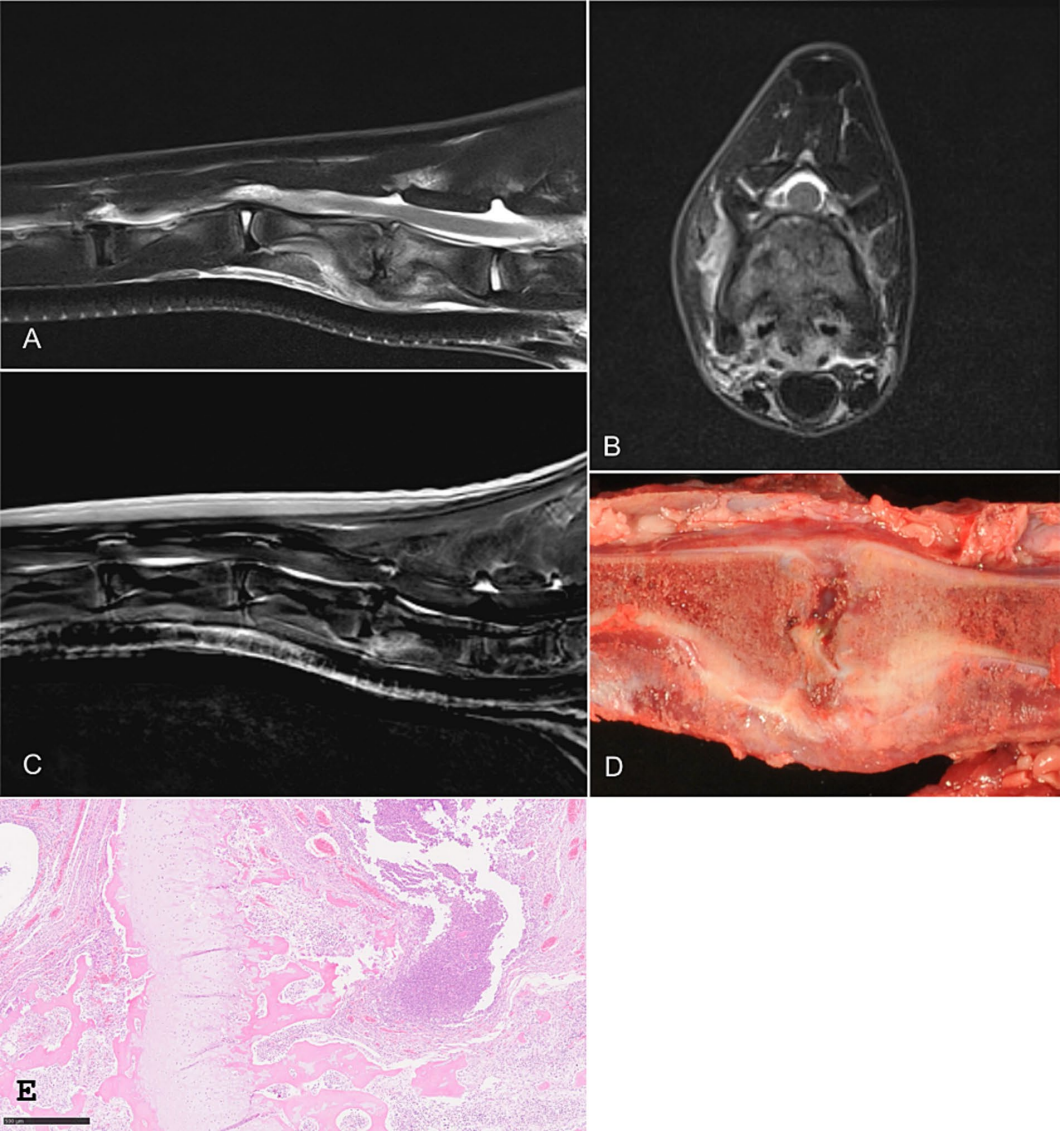
Sagittal T2-weighted **(A)**, transverse T2-weighted **(B)**, and post-contrast T1-weighted **(C)** MRI images of the cervical spine of one alpaca. Irregular endplates are visible between C5 and C6, with reduced signal intensity of the C5–C6 nucleus pulposus and mild disc protrusion. Hyperintense signal is present within the affected vertebrae, subvertebral tissues, and musculature. Mild contrast enhancement is visible in the cranial aspect of C6 and surrounding soft tissues. **(D,E)** Corresponding macroscopic and histopathologic sections. Severe chronic suppurative osteomyelitis with marked neutrophilic inflammation, granulation tissue formation, and reactive bone proliferation. MRI, magnetic resonance imaging.

Hematology and serum biochemistry were within normal limits, although serum amyloid A concentrations were high at 5.7 μg/mL (reference <2.7 μg/mL). An MRI of the cervical spine was performed with the cria positioned in lateral recumbency (Figure 1). The endplates between C5 and C6 displayed an irregular shape with decreased T2-signal from the nucleus pulposus of the intervertebral disc with mild disc protrusion. Ventral to the two affected vertebrae, heterogeneous T2- and short-TI inversion recovery (STIR)-hyperintense signals in the bones, musculature, and subvertebral tissue were present. Mild contrast enhancement was seen in T1-weighted images in the cranial aspect of C6 and the subvertebral musculature. Discospondylitis with associated paraspinal myositis was suspected.

Due to the severity of clinical findings and the progressive nature of the disease, the owners opted for humane euthanasia. Necropsy revealed severe chronic suppurative osteomyelitis with granulation tissue formation and extensive reactive bone proliferation between cervical vertebrae C5 and C6. These lesions were associated with perivascular hemorrhage in the grey matter of the cervical spinal cord.

##### 3.2.1.2 Case 2

A 2.5-year-old female alpaca was presented with a 3-week history of reduced general condition, diminished appetite, and intermittent recumbency. Two days prior to admission, the alpaca developed bruxism and a staggering gait with a lowered head posture. Clinical examination revealed marked cachexia, although vital parameters remained within normal reference limits. Orthopedic examination identified pronounced soreness over the thoracolumbar spine. Palpation of the neck and its flexion was unremarkable despite the lowered head posture. Neurological assessment demonstrated a non-ambulatory paraparesis with preserved spontaneous movement and deep pain perception but with loss of proprioception in the hind limbs, while forelimb function remained intact. Hyperreflexia of the patellar, flexor, and tibialis cranialis reflexes in both hind limbs suggested a lesion between the third thoracic and third lumbar spinal cord segments.

Despite impaired hindlimb function, the alpaca remained weight-bearing and assumed a compensatory posture in the pelvic limbs. Hematological analysis revealed leukocytosis at 26 × 10^9^/L (reference range: 8–21.4 × 10^9^/L) and mild anemia with a packed cell volume (PCV) of 0.2 L/L (reference range: 0.27–0.45 L/L). Blood chemistry and electrolyte levels were within the normal limits.

**Figure 2:**
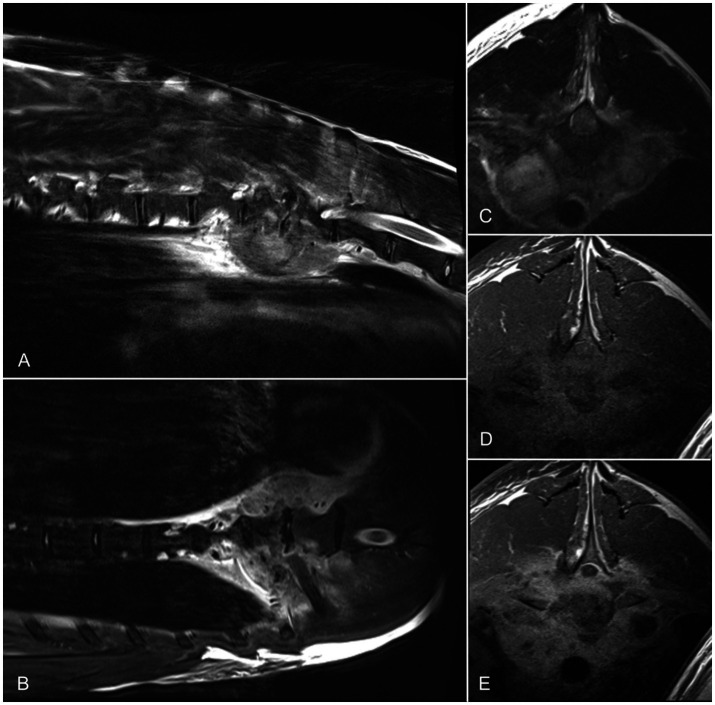
Sagittal **(A)**, dorsal **(B)**, and transverse **(C)** T2-weighted MRIs and pre- **(D)** and post-contrast **(E)** T1-weighted images of the thoracic spine of one alpaca. A poorly defined heterogeneous lesion is located ventral to Th9–Th11. The lesion is heterogeneously hyperintense on T2-weighted images and isointense on T1-weighted images, with intense peripheral contrast enhancement. The intervertebral space at Th9–Th10 is narrowed with reduced nucleus pulposus signal. Adjacent endplates show heterogeneous T2 hypointensity. Material within the spinal canal at Th10–Th11 shows mixed T1 and T2 signal and contrast enhancement, causing moderate dorsal compression of the spinal cord. MRI, magnetic resonance imaging.

Computed tomography and MRI of the spine were performed. Computed tomography revealed irregular osteolytic endplates of the adjacent Th10/Th11 intervertebral disc space and a paravertebral mass lesion with contrast enhancement at the same level. Additional MRI was performed in T1-W and T2-W sagittal, transverse, and dorsal images. The endplates of the caudal aspect of Th9 up to the cranial endplate of Th11, as well as the surrounding tissue, were hyperintense in T2 and STIR with irregular shape of the endplate Th10/11. A mass lesion was detected within the paravertebral muscles. Spinal cord compression was evident at the level of Th10/11 by T2-hyperintense material within the vertebral canal. Moderate contrast uptake was present in T1-weighted images within the endplates, vertebral canal and epaxial muscles. The diagnosis was discospondylitis with secondary spinal cord compression and the presence of a paravertebral abscess with secondary myositis of the epaxial musculature in Th9–Th11 (Figure 2).

The affected vertebral and paravertebral regions were punctured, and biopsy samples were submitted for microbiological analysis. Bacterial growth was not detected in the cultured material. Given the severity of the imaging findings and guarded prognosis, the owners elected humane euthanasia. Postmortem examination confirmed severe chronic purulent discospondylitis and osteomyelitis. Osteolysis and reactive bone proliferation were observed at thoracic vertebrae T10 and T11. Bacterial cultures remained negative.

##### 3.2.1.3 Case 3

A 1-year-old female llama was presented due to reduced general condition, disorientation, ataxia, and oral bleeding observed the day before admission. The animal had previously received antiparasitic treatment with toltrazuril (Baycox multi 50 mg/mL®, Elanco, Bad Homburg, Germany) at an oral dosage of 20 mg/kg.

At presentation, the llama was obtunded and showed severe ambulatory tetraparesis. Severe vestibular ataxia with a drift to the left affecting all four limbs was observed. Vital parameters were mildly altered, with a heart rate of 88/min (reference range: 48–80/min), respiratory rate of 36/min (reference range: 10–30/min), and body temperature of 37.6 °C (reference range: 37.5–38.9 °C). Mucous membranes were pink with mildly irregular episcleral vessels. Emergency hematological analysis revealed leukocytosis (27.4 G/L) (reference range: 8–21.4 × 10^9^/L), a mildly reduced PCV of 0.24 L/L (reference range: 0.27–0.45 L/L), and hyperglycemia (12.5 mmol/L) (reference range: 5.6–8.3 mmol/L). Electrolytes and acid–base status were within normal limits. Fecal parasitological examination revealed mild endoparasitosis, with 200 nematode eggs and 200 coccidian oocysts per gram of feces.

Following initial intravenous fluid therapy, the general condition of the llama stabilized, allowing for a complete neurological examination. The animal remained severely obtunded, with generalized vestibular ataxia and consistent left-ward drifting. Proprioception was severely reduced in all limbs. A left-sided head tilt, horizontal nystagmus with fast-phase saccades to the left, and right-sided facial paralysis were observed. Pupillary reflexes were bilaterally mildly decreased and no response was elicited on nasal septal stimulation. These findings suggested multifocal intracranial lesions involving the brainstem and forebrain.

**Figure 3:**
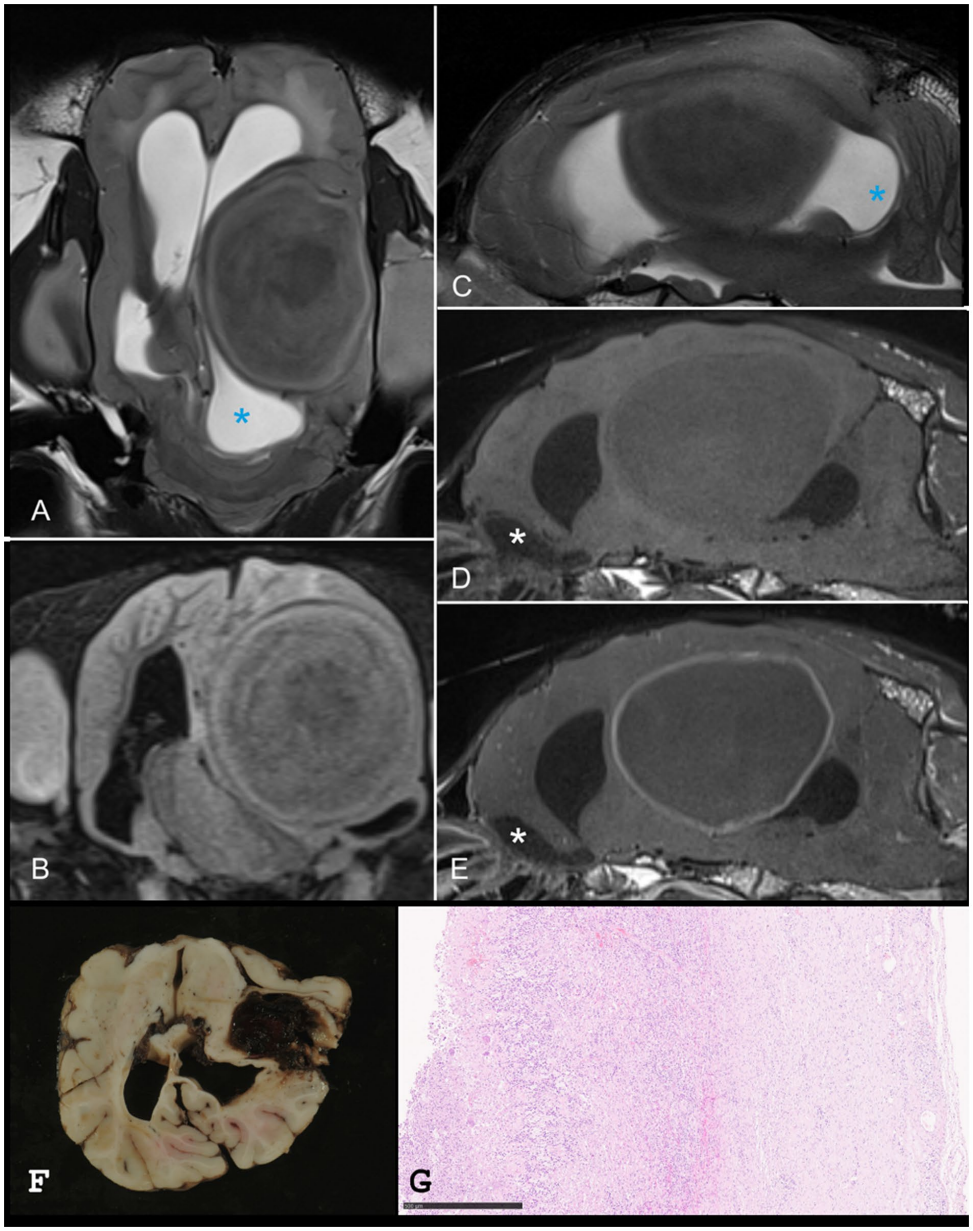
Dorsal **(A)** and parasagittal **(C)** T2-weighted images, transversal FLAIR **(B)**, and parasagittal pre- **(D)** and post-contrast **(E)** T1-weighted images of one dromedary camel. A well-defined, rounded intra-axial mass is present in the left temporal and parietal lobes. The lesion shows heterogenous T2 and FLAIR signals with concentric layers and a hypointense center. Perilesional hyperintensity is visible on T2 and FLAIR images. CSF outflow from the third ventricle is impaired, with secondary dilation of the lateral ventricles and olfactory recesses (blue and white asterisk) in both hemispheres. Panels F and G show corresponding necropsy and histopathology (HE, 7x, bar = 500 μm). CSF, cerebrospinal fluid; FLAIR, fluid attenuated inversion recovery.

Computed tomography and MRI of the head were performed. The llama was positioned in dorsal recumbency for imaging. Magnetic resonance imaging sequences included sagittal, transversal, and dorsal T2-W, transversal T2- FLAIR, and T1-W images before and after intravenous contrast administration (Figure 3). A focal, well demarcated, extra-axial mass lesion was observed at the level of the left temporal and parietal region. A large abscess was suspected in the left cerebral hemisphere resulting in a profound mass effect on the surrounding brain parenchyma with midline shift to the right. The abscess impaired CSF drainage from the third ventricle, resulting in moderate dilation of the lateral cerebral ventricles and olfactory recesses in both hemispheres. The caudal aspect of the left cerebral hemisphere expanded caudally causing compression of the cerebellum.

Despite the guarded prognosis, a left-sided craniotomy was performed to surgically remove the abscess. Computed tomography was performed to identify surgical landmarks as neurosurgery is not often performed on this species. Initially, the procedure progressed without complication. However, the patient developed intraoperative seizures that were unresponsive to therapeutic intervention and died under general anesthesia. Microbiological cultures of CSF and abscess material yielded no bacterial growth.

Postmortem examination revealed a large abscess in the left cerebral hemisphere, associated with malacia and extensive hemorrhage in the surrounding cerebral parenchyma. Moderate ventriculomegaly and subtentorial herniation of the occipital lobe were also present. Histopathological evaluation of the cerebral lesion confirmed chronic suppurative inflammation, characterized by purulent exudate, granulation tissue formation, and multifocal infiltration with foreign body-type multinucleated giant cells, and no microorganisms were found. Sparse birefringent material was observed within the cellular debris and pus, presumed to be exogenous contamination. This interpretation was supported by the absence of bacterial growth in culture.

##### 3.2.1.4 Cases 4 and 5 - otitis media and Interna in two alpacas

A 14-month-old female alpaca was referred and had previously been diagnosed with chronic otitis, characterized by purulent discharge from the right external acoustic meatus. Pretreatment included local antibiotic ear flushing and direct bulla tympanica instillation of enrofloxacin, informed by antimicrobial resistance testing and CT at a private Center for Small Animal Medicine. The animal had also been treated with ivermectin and vaccinated against *Clostridium* spp. (Covexin zehn®; Zoetis, Berlin, Germany).

On presentation, the alpaca was presented with a body condition score 4/9, weighing 33.2 kg. Vital parameters were mildly increased due to excitement (heart rate 68/min, respiratory rate 48/min, and temperature 38.1 °C). The neurological examination was unremarkable. However, purulent discharge was visible in the right external ear canal. Bacterial swabs were collected and endoscopic examination following sterile saline lavage revealed necrotizing otitis externa.

Microbiological analysis revealed the presence of *Bacillus* spp., *Enterococcus gallinarum,* and *Rhodococcus hoagie*, with *Trueperella pyogenes* identified as the predominant pathogen.

Due to the chronicity of the condition, follow-up CT imaging was recommended, including contrast administration. The animal was positioned in lateral recumbency and scanned under general anesthesia. Computed tomography confirmed previous findings: the right external acoustic meatus, tympanic bulla, and inner acoustic meatus were filled with heterogeneously soft tissue-attenuating material. The right bulla tympanica was severely deformed with poorly defined trabeculae and indistinct osseous margins, particularly ventrally and laterally. The medial bulla wall was thickened and showed multiple osteolytic lesions. Post-contrast imaging demonstrated marked enhancement of the bulla lumen and osseous remnants, while the external acoustic meatus contents showed no enhancement. The right medial retropharyngeal lymph node was moderately enlarged.

**Figure 4:**
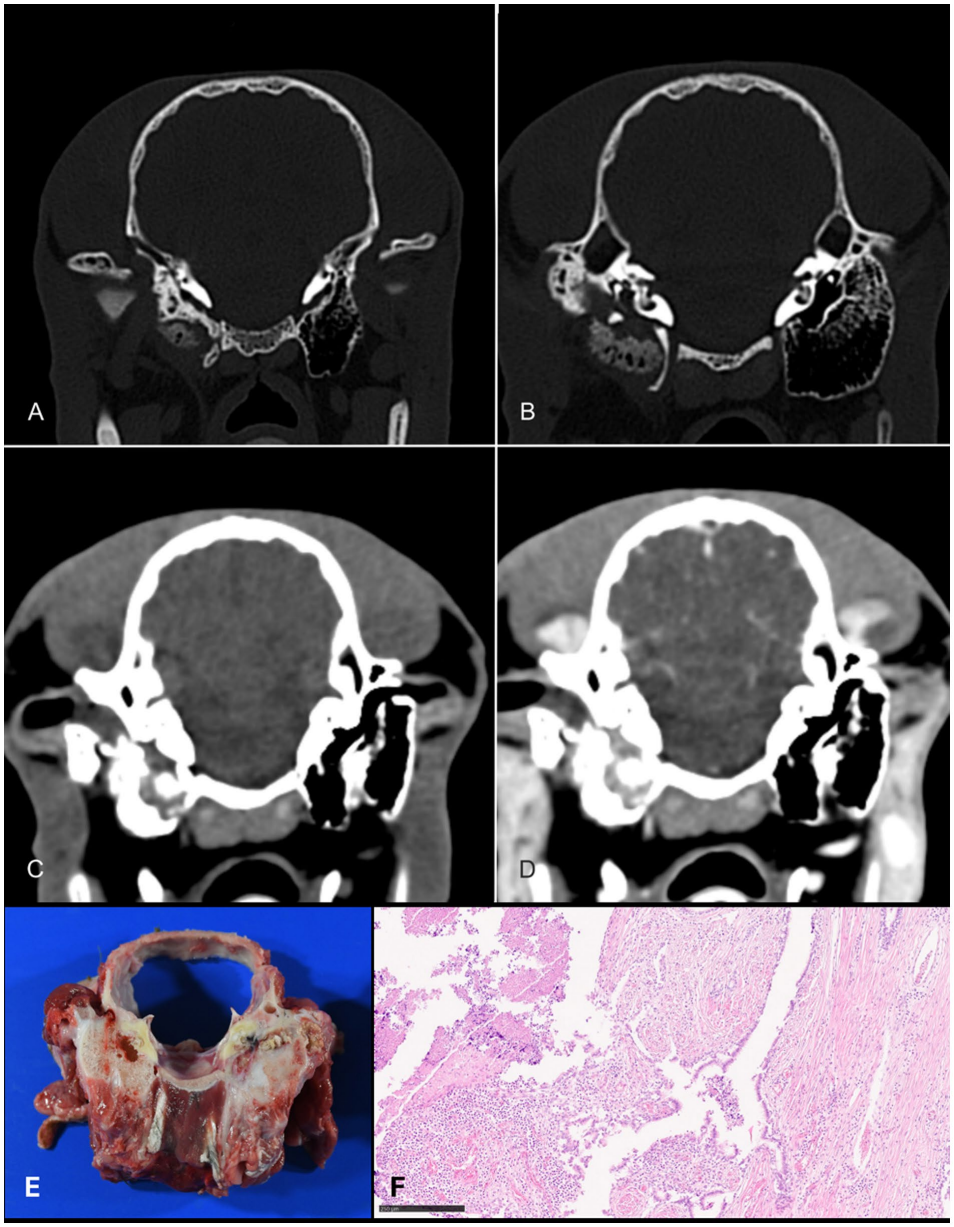
Transversal CT images in the bone **(A,B)** and soft tissue **(C,D)** windows of one alpaca acquired before **(C)** and after **(D)** contrast administration. The osseous external auditory canal is enlarged and exhibits multifocal osteolysis. The bulla tympanica shows loss of trabecular structure and contains soft tissue-attenuating material. After contrast administration, enhancement is visible within the tympanic cavity. (E, F) Corresponding necropsy and histopathology findings confirming chronic suppurative otitis with bulla empyema and loss of trabecular bone (HE, 10×, bar = 250 μm). CT, computed tomography.

These findings supported the diagnosis of advanced right-sided chronic otitis externa and media, with secondary hyperostosis of the *os tympanohyoideum* (Figure 4).

In consultation with the owners, surgical intervention was pursued via lateral bulla osteotomy as previously described (12). Postoperatively, the alpaca showed severe ambulatory tetraparesis but exhibited severe torticollis and rightward head tilt, with mild positional rotatory nystagmus in both eyes. Pain management included a continuous intravenous infusion of butorphanol (0.03 mg/kg/h; Torbugesic®; Zoetis), lidocaine (30 μg/kg/min; Lidocainhydrochlorid 2%®; Bela-Pharm GmbH, Vechta, Germany), and ketamine (10 μg/kg/min; Ketamin 100 mg/mL®; CP-Pharma, Burgdorf, Germany). Metamizole (40 mg/kg; Metamizol WDT®; WDT, Garbsen, Germany) was administered intravenously three times daily. Antibiotic therapy included oxytetracycline (20 mg/kg; Ursocyclin®; Serumwerk, Bernburg, Germany).

Despite initial improvement, the general condition of the alpaca deteriorated over the following days. It became recumbent, anorexic, hyperthermic (up to 42 °C), and showed severe sigmoid torticollis. When the torticollis was manually corrected, the alpaca showed severe ambulatory tetraparesis, accompanied by urination and defecation. Use of a supportive ruff induced labored open-mouth breathing. The animal died 8 days post-surgery and underwent necropsy, which confirmed chronic suppurative otitis media with dorsal bulla empyema (Figures 4E,F). The trabecular structure of the bulla was absent. There was no evidence of cranial or intracerebral penetration by the infection.

##### 3.2.1.5 Case 5

The second case of otitis was observed in a 3-year-old Huacaya alpaca gelding. The animal presented with decreased activity, self-imposed isolation, frequent recumbency, and reduced appetite and defecation for the last day prior to referral. On initial examination, the alpaca presented a reduced general body condition, showing stuporous mentation, dorsal rotation of the right bulbus, and tachypnea (respiratory rate of 60/min). Other vital parameters were within normal limits.

Neurological examination revealed right-sided pleurothotonus, non-ambulatory tetraparesis, and reduced proprioception. Palpebral reflex and menace response were absent bilaterally. Bilateral jerk nystagmus with a fast phase to the left was observed, changing to positional vertical nystagmus when the neck was flexed dorsally. Miosis, ptosis, and enophthalmos, consistent with Horner syndrome, were present on the right side. Based on these findings, a brainstem lesion with right-sided lesion of the central vestibular system was suspected.

**Figure 5:**
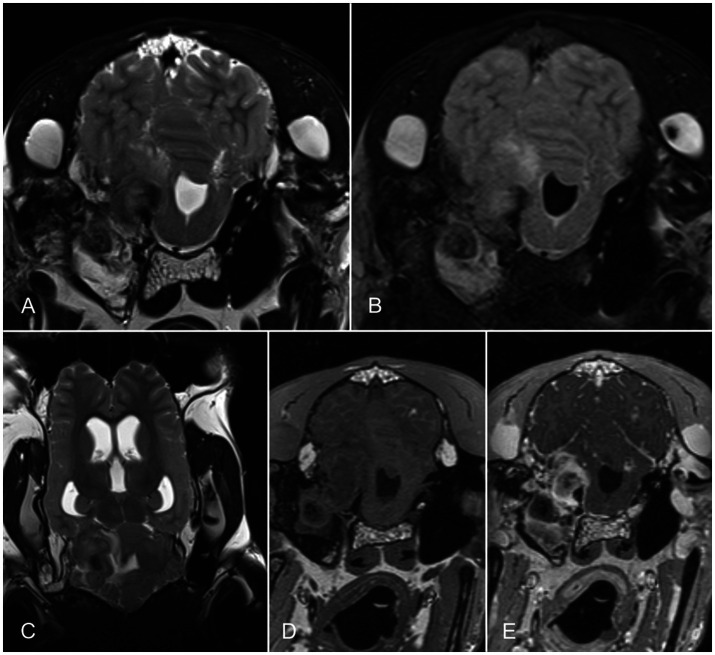
Transversal T2-weighted **(A)**, FLAIR **(B)**, dorsal T2-weighted **(C)**, pre-contrast T1-weighted **(D)**, and post-contrast T1-weighted **(E)** MRI of one alpaca. The right external ear canal shows a thickened, hyperintense wall. The right tympanic bulla is widened and filled with heterogenous material. A well-defined ovoid lesion is visible adjacent to the petrous bone defect, extending into the cranial vault. The lesion demonstrates heterogenous FLAIR and T2 signal, susceptibility artifacts, and peripheral contrast enhancement. Mass effect is present on the cerebellum and brainstem. Ventricles are moderately enlarged, with narrowing of the subarachnoid space at the foramen magnum. CT, computed tomography; fluid attenuated inversion recovery; MRI, magnetic resonance imaging.

The alpaca underwent an MRI of the head in sternal recumbency (Figure 5).

Magnetic resonance imaging revealed severe right-sided T2- and FLAIR hyperintense heterogeneous material within the external ear canal, middle ear cavity and inner ear with intracranial extension and formation of an extra-axial mass lesion at the level of the right *os petrosum*. Additional findings included moderate adjacent intra-axial T2 and FLAIR hyperintense signal within the cerebellum consistent with edema, and secondary ventriculomegaly with signs of increased intracranial pressure. Focal contrast enhancement in T1-weighted images was present within the adjacent meninges. Erosive arthritis of the right temporomandibular joint, widening of the right dorsal subarachnoid space, and signal changes in the medulla oblongata were also observed. Based on imaging, chronic bacterial otitis externa, media, and interna with otogenic abscess formation extending into the neurocranium and right temporomandibular joint was suspected. Due to the poor prognosis, the owners elected euthanasia. Necropsy was declined.

##### 3.2.1.6 Case 6 - intracranial mycotic granuloma formation in a dromedary

An 11.75-year-old female dromedary presented with a 3-week history of progressive gait and behavioral abnormalities.

Vital parameters, heart rate, and body temperature could not be obtained due to persistent movement. The respiratory rate was 24/min. Mucous membranes were mildly cyanotic, and the jugular veins appeared normal on palpation. The gastrointestinal compartments were moderately filled with altered layering. The abdominal wall was relaxed, and fecal consistency was normal.

Upon clinical examination, the dromedary was recumbent in sternal position and obtunded. It was presented with a body weight of approximately 730 kg and a body condition score 2/9. The animal displayed fluctuating behavior ranging from apathy to excitation. When standing, it showed its head pressing against the wall, circled to the right, and exhibited right-sided pleurothotonus. Due to uncooperative behavior, proprioceptive deficits could not be assessed. Cranial nerve reflexes were within normal limits. A right forebrain lesion was suspected based on behavioral and postural signs.

Because of persistent neurological symptoms, an MRI of the head was pursued. Sedation was achieved using xylazine (0.1 mg/kg Proxylaz ®; Veyx-Pharma GmbH, Schwarzenborn, Germany), and a permanent jugular vein catheter (Infusion catheter set®; G-14, Walter Vet Instruments e.K., Baruth / Mark; Germany) was placed to intravenously administer isotonic sodium chloride (0.9% NaCl, Serumwerk Bernburg AG) and dexamethasone (0.06 mg/kg, Dexamethason 4 mg/mL®, Bela-Pharm GmbH). The animal was stabilized and underwent a second neurological assessment.

**Figure 6:**
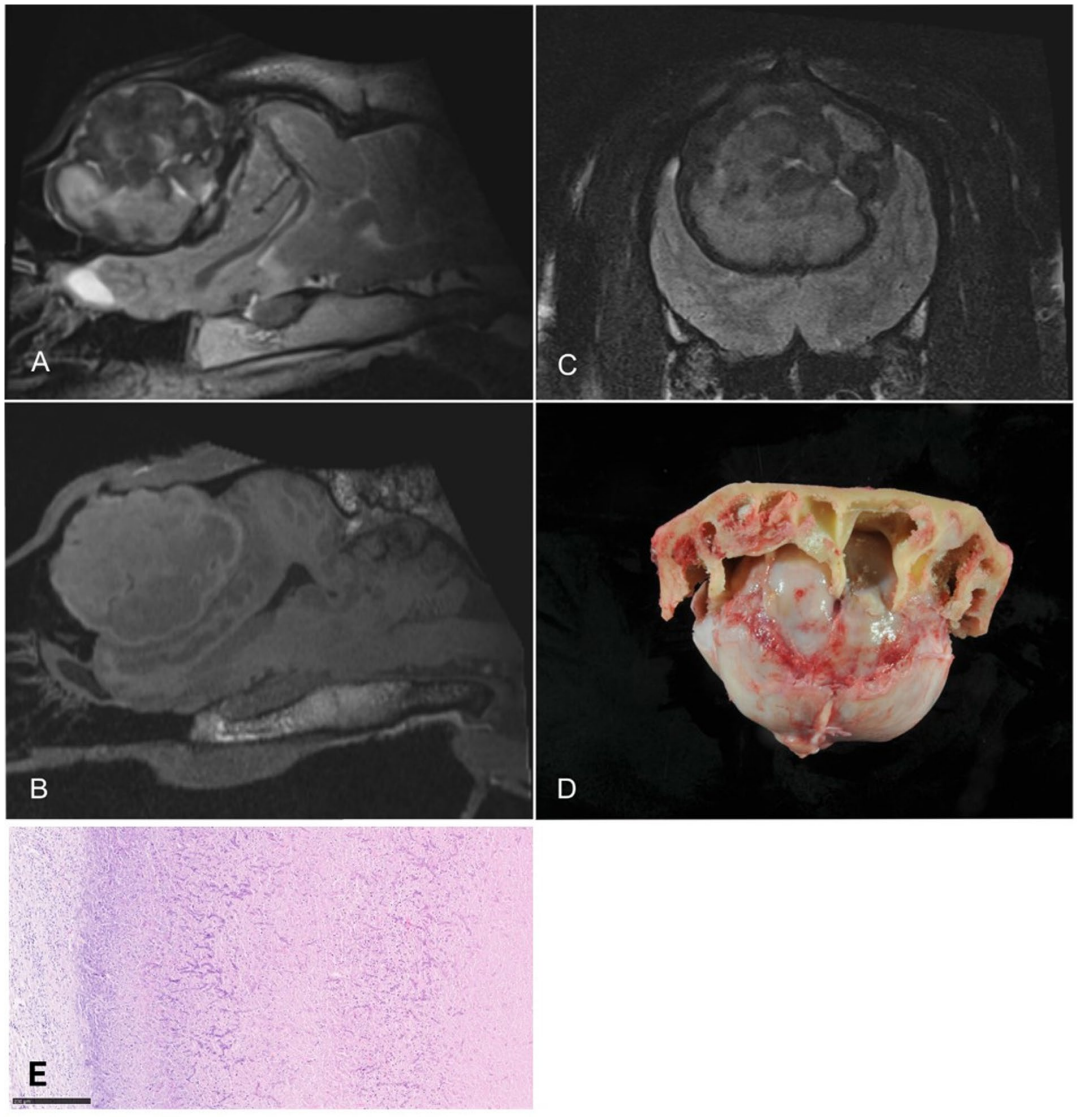
Sagittal **(A)** and sagittal **(B)** T1- and transversal T2-weighted **(C)** images of one dromedary camel. A centrally located, rounded heterogeneous mass extends into the frontal sinus and exerts marked mass effect on the forebrain **(D)**. No contrast was administered. **(E)** Histopathology (HE, 10x, bar = 250 μm) corresponding to the intracranial mass.

An MRI was performed under general inhalation anesthesia. Sagittal, transverse, and dorsal T2-W images and sagittal T1-W sequences were acquired in lateral recumbency (Figure 6). A focal, well demarcated, space-occupying, rounded, heterogeneous, extra- axial mass was located ventral and caudal to the frontal sinus with extension into the cranial cavity, resulting in a severe mass effect on the entire forebrain. The occipital lobes were displaced into the caudal cranial fossa, compressing the midbrain. The mass had T2- and T1-hyper- and hypointense regions of varying dimensions. Contrast administration was omitted due to financial constraints. Differential diagnoses included granuloma or neoplasia.

Given the poor prognosis, the owner requested euthanasia and authorized a postmortem examination. Cerebrospinal fluid collected from the lumbar region postmortem showed no cytologic abnormalities. Necropsy revealed a well-circumscribed, partially mineralized mass measuring approximately 7 cm in diameter, causing marked compressive atrophy of both frontal lobes. Histological analysis demonstrated a granulomatous inflammatory process with extensive necrosis and dense aggregates of fungal hyphae, consistent with a mycotic granuloma (11). Microbiological examination of the lesion identified low levels of *Aspergillus fumigatus*, as well as alpha-hemolytic *Streptococci*, *Serratia marcescens*, and *Rothia nasimurium,* confirming a fungal granuloma with secondary bacterial infection.

#### 3.2.2 Group B: traumatic disease of the spine

##### 3.2.2.1 Case 7

A 3-year-old male llama presented with a history of ataxia, which had shown transient improvement following corticosteroid therapy. Antimicrobial treatment had also been initiated by the referring veterinarian, who unsuccessfully attempted CSF collection at the L5/6 intervertebral space. The animal was referred for further evaluation. General clinical examination findings and vital parameters were within normal limits. Neurological examination demonstrated proprioceptive hindlimb ataxia, slightly reduced proprioception in both hindlimbs, and mildly decreased patellar reflexes bilaterally. A spinal cord lesion was suspected at the level of the fourth to sixth lumbar segment. Hematological analysis revealed moderate anemia, with a PCV of 0.19 L/L, and hyperglycemia (15.2 mmol/L).

**Figure 7:**
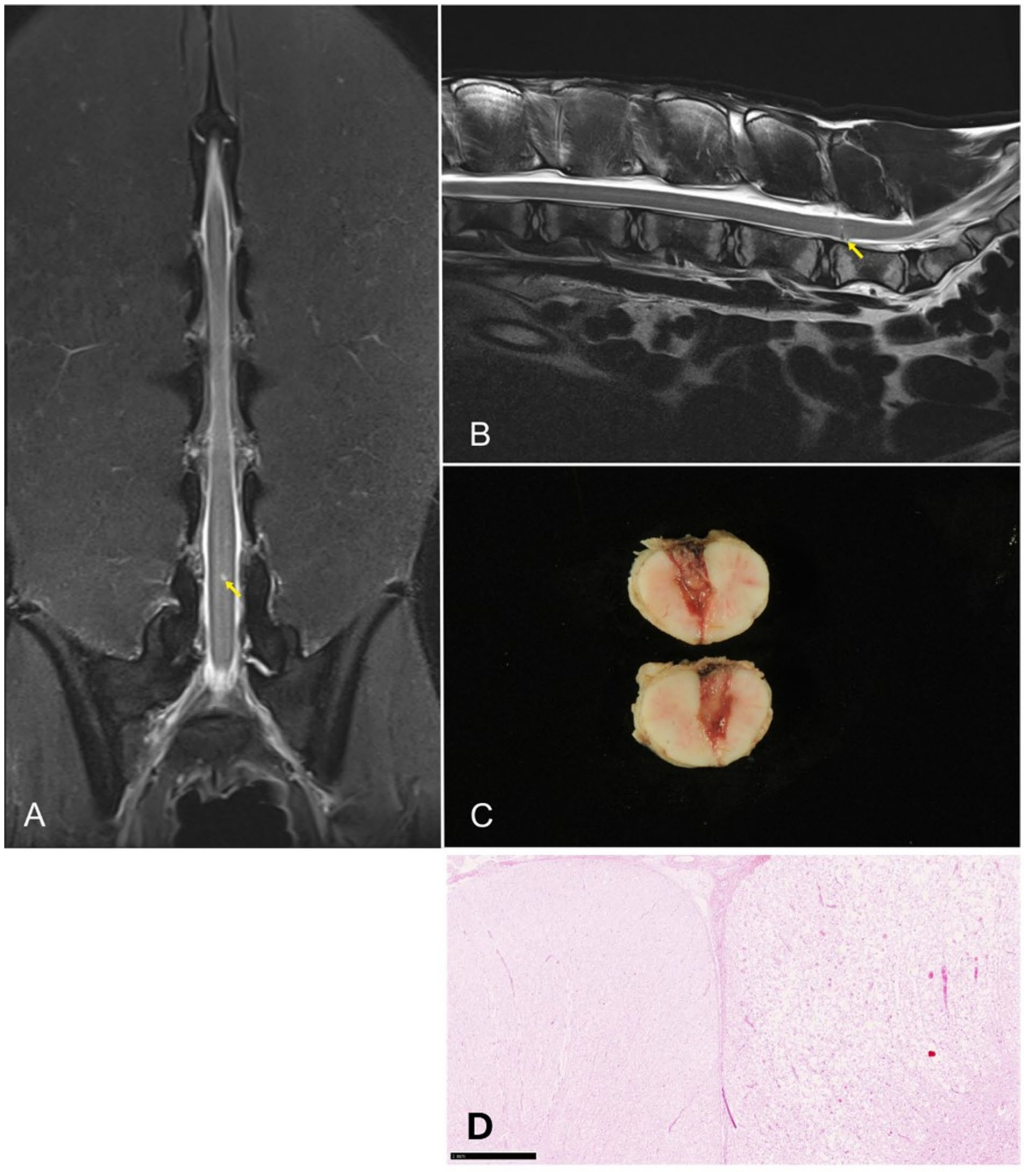
Dorsal **(A)** and sagittal **(B)** T2-weighted images of the lumbar spine of one llama. A small, ill-defined intramedullary hyperintense lesion is present at L5–L6 (yellow arrows). **(C)** Transverse section of the spinal cord displaying a large wedge-shaped lesion of the myelon. **(D)** Histopathologic section of the spinal cord (HE, 2.5x, bar = 1 mm).

Following discussion with the owner, an MRI of the spine was performed. The animal was placed in dorsal recumbency. Imaging included dorsal and sagittal STIR, sagittal and transverse T2-W sequences, and sagittal and transverse T1-W sequences, with and without contrast administration. MRI findings are presented in Figure 7. At the level of L5/L6, a small, ill-defined, straight intramedullary T2-hyperintense lesion was visible. An iatrogenic spinal cord injury was diagnosed following attempted CSF collection. In the days following imaging, gait abnormalities worsened, and the animal became paraparetic 6 days later. Due to the progressive clinical deterioration, the owners elected euthanasia. Necropsy revealed a wedge-shaped lesion extending across nearly the entire diameter of the caudal lumbar spinal cord. Histopathological analysis confirmed focal myelomalacia with associated edema, hemorrhage, and phagocytic removal of necrotic debris. The cause for the reported ataxia before CSF examination remains unclear in this case. A preexisting spinal cord lesion, such as ischemic infarction of the spinal cord, could be a reason for the reported ataxia. However, this was not confirmed in MRI or pathological examination.

##### 3.2.2.2 Case 8

**Figure 8:**
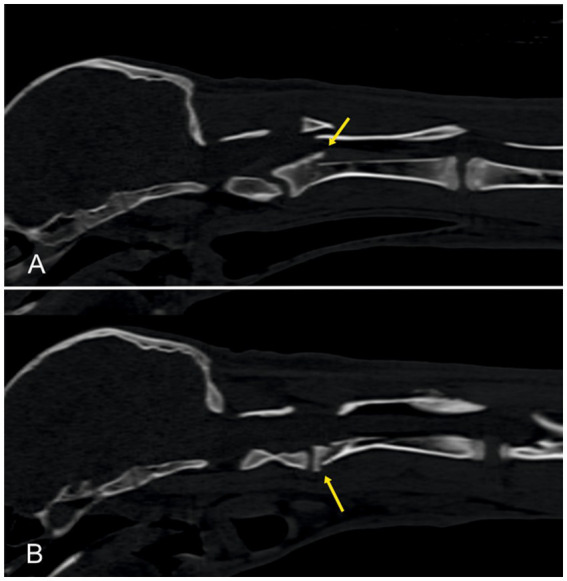
Sagittal reformatted CT images of the cervical spine of one alpaca. An oblique fracture line is visible through the cranial endplate of the axis with dorsal displacement of the fracture fragment and a step formation within the spinal canal, resulting in spinal cord compression (yellow arrows). CT, Computed tomography.

A 6-month-old male Huacaya alpaca presented with a cervical injury after being trapped in a pasture fence. At the time of admission, the animal was recumbent and apathetic. It displayed tachypnea, with a respiratory rate of 48 breaths/min, while other vital parameters were within normal limits. Vision and cranial nerve reflexes were preserved. Neurological examination revealed non- ambulatory tetraparesis with increased muscle tone and mildly exaggerated reflexes in all four limbs. A presumptive diagnosis of cervical spinal injury (C1–C5) was made. The alpaca was transferred for CT imaging of the caudal cranium and cervical spine, performed in dorsal recumbency. Computed tomography revealed a fracture through the cranial endplate of the second vertebra with a dorsal displacement of the caudal fragment and a secondary step formation within the spinal canal, resulting in severe spinal cord compression (Figure 8). Due to the poor prognosis, the owners elected euthanasia. Postmortem examination was declined.

#### 3.2.3 Group C: congenital diseases – case 9

**Figure 9:**
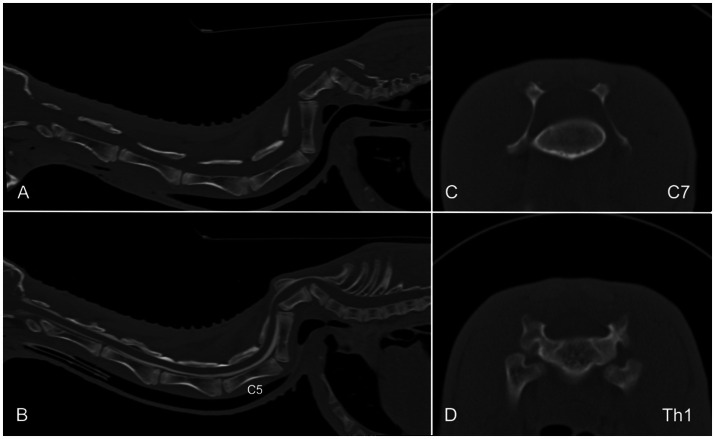
Sagittal reformatted CT images of the cervical and cranial thoracic spine of one alpaca before **(A,C,D)** and after myelographic contrast injection **(B)**. Severe kyphosis of the lower cervical spine is evident. C7 and Th1 are shortened and wedge-shaped, with absent vertebral arches. A dose of 0.3 mL/kg iodine contrast medium was administered into the cisterna magna. Contrast flow is reduced at C5 and intermittently visible at C7, with loss of the ventral column at caudal C6. CT, computed tomography.

A 4-month-old female alpaca was presented with slowly progressive gait abnormalities affecting all four limbs. General examination was unremarkable. Neurological examination revealed moderate ambulatory tetraparesis with proprioceptive deficits in all limbs, while spinal reflexes remained normal. A pronounced spinal deviation and kyphosis of the lower cervical spine was noted on palpation. Neuroanatomical localization was C6–Th2. Hematology and serum biochemistry results were within normal limits. CT of the head and spine was performed in lateral recumbency (Figure 9), showing a severe kyphosis of the lower cervical vertebral column. C7 and Th1 were shortened in length, and the rostral extremity was wedge-shaped, causing malarticulation with the cranial vertebral segment, which resulted in subluxation and narrowing of the vertebral canal. The vertebral arches of C7 and Th1 were absent. A cervical CT myelography was performed by administration of 0.3 mL/kg iodine contrast agent (Omnipaque ®; GE Health Care) into the cisterna magna. Contrast passed readily from the cisterna magna until C5. Over C5, the dorsal contrast column was severely diminished, reappearing for a short distance over C7. At the caudal aspect of C6, the ventral contrast column was no longer visible and the dorsal column reappeared. No other skeletal malformations were observed. A diagnosis of congenital spinal malformation with compression of the lower cervical spinal cord was made. The owner declined further treatment, and the alpaca was discharged.

#### 3.2.4 Group D: normal findings

Normal findings were observed in the brain (*n* = 4), cervical spine (*n* = 1), thoracic spine (*n* = 2), and lumbar spine (*n* = 3). These animals were either diagnosed with neurological conditions not associated with the brain (e.g., discospondylitis with myelocompression) or underwent imaging of multiple regions, resulting in normal and abnormal findings within the same animal.

Clinical signs among these cases included head tilt, nystagmus, trauma-associated intraocular hemorrhage, paresis or ataxia, and various forms of lameness. Two animals were referred for diagnostic imaging due to ataxia or lameness suspected of having conditions of a neurological origin but were ultimately diagnosed with orthopedic disorders (e.g., arthrosis). One alpaca presented with myiasis and widespread dermatitis accompanied by a head tilt. An MRI was performed to rule out otitis. The head tilt was attributed to myiasis-associated myositis secondary to severe dermatitis and was not neurologically derived. The underlying dermatological conditions were treated and the animal was discharged.

## 4 Discussion

Neurological diseases in camelids are relatively common, but diagnosis in this species remains challenging compared to that of companion animals. These challenges stem from differences in behavior, temperament, housing, and the distinct nature of owner-animal interactions. Behavioral changes, altered food intake, and postural abnormalities are readily recognized by owners of traditional companion animals, such as dogs and cats. In contrast, camelids tend to be shy and have only recently undergone selective breeding for closer human interaction (13). As a result, camelids are often presented to veterinary clinics at a later stage of disease, frequently exhibiting only subtle clinical signs despite the presence of significant underlying pathology.

In addition to neurological examinations (14), CSF analysis and diagnostic imaging are recommended for animals with suspected neurologic conditions (2). However, owner compliance with such recommendations is frequently limited. Decisions to pursue diagnostic workup are often influenced by cost considerations and the perceived futility of treatment, particularly when a severe or untreatable condition is suspected. To the best of our knowledge, our study represents the first reported case series of tylopods undergoing MRI or CT imaging and demonstrates that most cases had a poor prognosis, with most animals either euthanized or dying shortly after diagnosis.

Clinicians also face significant technical constraints, particularly related to the size and body weight of camelids. Adult alpacas and llamas can weigh up to 90 and 250 kg (13), respectively, which can limit the feasibility of full-body imaging. However, the anatomical features of SACs, including their relatively small heads and elongated necks, are favorable for targeted imaging of the cranial and cervical regions. Still, MRI and CT of the spinal cord is generally limited to young or smaller animals, introducing a potential bias in the published literature on spinal cord pathologies in adult camelids.

Another critical consideration when performing MRI and CT in SACs is the variation in drug and livestock legislation depending on the country wherein examinations are performed. In Germany, no drugs are licensed specifically for use in SACs, requiring the off-label administration of all medications in this species. Because SACs are classified as livestock and may be designated for meat production, certain drugs are prohibited, including contrast media. This further limits the diagnostic options available, particularly in animals intended for food production. An additional challenge is the correct positioning of these animals during MRI or CT. While sternal recumbency is considered the safest position for ruminants under general anesthesia due to its reduced cardiopulmonary compromise (15), the species-specific anatomy of SACs, particularly the large triangular chest and long mobile neck, led to significant respiratory motion artifacts. These movements, associated with inspiration and expiration, interfered with stable head positioning. Attempts to fix the head within the coil were unsuccessful. For brain studies, right lateral recumbency was used in most cases, whereas imaging of the spine required dorsal recumbency to achieve appropriate vertebral alignment.

Currently, MRI/CT findings of systemic infections, or meningoencephalitis regardless of their origin, have not been described in SACs. In small ruminants, such as sheep and goats, the most common causes of neurological disease are infectious or toxometabolic in origin (16, 17).

Accurate diagnosis of systemic neurological diseases requires detailed species-specific knowledge of MRI brain anatomy (18, 19). While the brain anatomy of sheep and goats has been documented (18, 20, 21), similar anatomical data for SACs remain limited, complicating the diagnostic process. Future research should aim to clarify the normal and abnormal neuroanatomy of SACs and address comparative neuroimaging characteristics in this species.

Most of MRI and CT findings in the present case series involved inflammatory conditions, including otitis, brain abscesses, and discospondylitis. These findings are consistent with existing case reports and may support the notion that SACs might be particularly prone to focal, inflammatory neurological diseases (6, 10, 12, 22–27). Among the 14 published reports describing neurologic disorders in SACs, the distribution of conditions was approximately 35.7% otitis (*n* = 5), 28.6% discospondylitis (*n* = 4), 21.4% abscess or cyst formation (*n* = 3), and 14.2% congenital malformation or trauma (*n* = 2). These frequencies align with the cases described in our study, suggesting that our data may reflect the broader global distribution of neurological disorders in SACs. Microbiological culture of the sampled discospondylitis cases was positive in only one previously published case (10), which showed a polymicrobial infection, including high levels of *α*-hemolytic *Streptococcus*, mild counts of *Corynebacterium* spp., and *Staphylococcus epidermidis*, and low counts of *Alcaligenes faecalis*, *γ*-hemolytic *Streptococcus*, and *Proteus* spp.

Fungal infections in camelids are most commonly associated with respiratory diseases (28) or cutaneous mycoses (29). A single case report described scrotal granulomatous aspergillosis in a dromedary camel, presenting as a subcutaneous mass (30). The macroscopic and histopathological features resembled those observed in our case of suspected intracranial fungal granuloma. To the best of our knowledge, CNS aspergillosis has not previously been documented in camelids. In the present case, coinfection with *A. fumigatus* and *Rothia nasimurium* is presumed to have occurred secondary to immunosuppression. Although no comorbidities were reported in this case, immunosuppression is likely in animals with fungal infections according to the literature and therefore must be suspected here despite the lack of history (31). The most likely route of infection was ascending invasion from the oronasal cavity.

Congenital malformations of the spine or CNS have been reported in both crias (32) and adult alpacas (33). Trauma-related conditions, such as cervical spinal injuries, are relatively common in SACs (5), likely due to aggressive behaviors involving the head and neck, particularly in males (13). However, luxation or subluxation of spinal vertebrae has rarely been described (34) and was observed in only two cases in our study.

In three cases in our study, MRI or CT findings were unremarkable despite the presence of clinically evident neurologic signs. Gait disturbances, postural abnormalities, and head tilt were initially attributed to CNS involvement; however, diagnostic imaging ruled out CNS pathology. These cases demonstrate that clinical examination alone may not be sufficient to reliably distinguish between neurological and orthopedic conditions. In some instances, abnormal behaviors may mimic neurologic signs and lead to diagnostic confusion. The aforementioned three cases were diagnosed as osteophytic arthrosis of cervical vertebrae with sequester formation and myositis of cervical musculature, myiasis associated alteration of behavior, and gonarthritis, respectively. Some inherent limitations of this study due to the retrospective design are worth mentioning. The main limitation is the paucity of information on clinical and neurological examinations, particularly in older cases. Documentation in more recent cases is more specific, and the examiners are more experienced due to an increasing number of SACs being presented at the clinic. However, the character and the compliance behavior of the animals often cause difficulties in performing thorough clinical neurological examinations. Therefore, in some instances, inappropriate regions were scanned, or imaging was unnecessary because the defects were not localized to the CNS.

Another consequence of the retrospective study design is that CT/MRI protocols are not standardized but adjusted to each animal’s specific clinical findings. Furthermore, not all animals underwent necropsy. Therefore, confirmation of diagnostic imaging findings was not available for all animals in the study.

In conclusion, advanced diagnostic imaging is crucial in the diagnostic workup of presumed neurological disease in camelids. This study highlights the broad spectrum of conditions associated with neurological symptoms in SACs. However, due to the complexity of neurological evaluation in camelids, clinical signs suggestive of neurological disease may be associated with normal CT and MRI findings because of concurrent non-neurological conditions. Additionally, normal MRI or CT findings do not necessarily exclude the presence of neurologic disease.

## Data Availability

The data underlying this article cannot be shared publicly due to the privacy of animal owners. The data will be shared on reasonable request to the corresponding author. Requests to access these datasets should be directed to Marlene.Sickinger@vetmed.uni-giessen.de.
